# Derivation and Clinical Utility of Safety Targets for Linezolid-Related Adverse Events in Drug-Resistant Tuberculosis Treatment

**DOI:** 10.3390/ph16111575

**Published:** 2023-11-08

**Authors:** Lina Keutzer, Laurynas Mockeliunas, Marieke G. G. Sturkenboom, Mathieu S. Bolhuis, Onno W. Akkerman, Ulrika S. H. Simonsson

**Affiliations:** 1 Department of Pharmaceutical Biosciences, Uppsala University, 751 24 Uppsala, Sweden; 2 Department of Clinical Pharmacy and Pharmacology, University Medical Center Groningen, University of Groningen, 9713 GZ Groningen, The Netherlands; 3 Department of Pulmonary Diseases and Tuberculosis, University Medical Center Groningen, University of Groningen, 9713 GZ Groningen, The Netherlands; 4 Tuberculosis Center Beatrixoord, University Medical Center Groningen, University of Groningen, 9751 ND Groningen, The Netherlands

**Keywords:** tuberculosis, pharmacokinetics, linezolid, Monte Carlo simulation, indirect response modelling, time-to-event analysis, safety, peripheral neuropathy, myelosuppression, clinical utility index

## Abstract

Long-term usage of linezolid can result in adverse events such as peripheral neuropathy, anemia and thrombocytopenia. Therapeutic drug monitoring data from 75 drug-resistant tuberculosis patients treated with linezolid were analyzed using a time-to-event (TTE) approach for peripheral neuropathy and anemia and indirect response modelling for thrombocytopenia. Different time-varying linezolid pharmacokinetic exposure indices (AUC_0–24h,ss_, C_av_, C_max_ and C_min_) and patient characteristics were investigated as risk factors. A treatment duration shorter than 3 months was considered dropout and was modelled using a TTE approach. An exposure–response relationship between linezolid C_min_ and both peripheral neuropathy and anemia was found. The exposure index which best described the development of thrombocytopenia was AUC_0–24h_. The final TTE dropout model indicated an association between linezolid C_min_ and dropout. New safety targets for each adverse event were proposed which can be used for individualized linezolid dosing. According to the model predictions at 6 months of treatment, a C_min_ of 0.11 mg/L and 1.4 mg/L should not be exceeded to keep the cumulative probability to develop anemia and peripheral neuropathy below 20%. The AUC_0–24h_ should be below 111 h·mg/L or 270 h·mg/L to prevent thrombocytopenia and severe thrombocytopenia, respectively. A clinical utility assessment showed that the currently recommended dose of 600 mg once daily is safer compared to a 300 mg BID dosing strategy considering all four safety endpoints.

## 1. Introduction

Rifampicin-resistant, including multidrug-resistant, tuberculosis (MDR-TB) is a threat to global health, presented by roughly 450,000 patients in 2021 [1]. Linezolid is part of the recommended treatment regimens against MDR- and XDR-TB and has proven its efficacy in clinical trials such as the Nix-TB trial [2], the ZeNix trial [3] and the TB-PRACTECAL trial [4]. The findings of these clinical trials were adopted into the WHO guidelines for the treatment of drug-resistant tuberculosis (TB) in 2022 [5]. The first choice is the 6 month all-oral BPaLM regimen consisting of bedaquiline, pretomanid, linezolid and moxifloxacin [5]. The second choice is a 9 month all-oral regimen and the third choice is a regimen of 18 months or longer [5]. Linezolid toxicity is of concern, especially after long-term treatment (longer than 28 days), as is the case with TB treatment [5]. Myelosuppression, irreversible peripheral neuropathy and optic neuritis are severe adverse events that have been observed following long-term linezolid treatment [2,6,7]. These are suspected to be related to mitochondrial toxicity [8,9,10]. Linezolid is thought to inhibit human mitochondrial ATP synthesis by binding to the 16S rRNA subunit [9,10]. In the Nix-TB trial, linezolid was studied at a dose of 1200 mg daily for up to 26 weeks, where 81% of the patients experienced peripheral neuropathy and 48% myelosuppression [2]. In an effort to reduce linezolid toxicity, the ZeNix trial was launched in 2017, investigating shorter treatment length and/or lower linezolid doses combined with bedaquiline and pretomanid [3]. The results suggest that reducing the dose to 600 mg once daily (QD) leads to increased safety, while maintaining efficacy [3].

Efforts have been made to identify a safety target to reduce the risk of developing any of the above-mentioned severe adverse events. Toxicity (myelosuppression, optic neuritis and peripheral neuropathy) has been shown to be correlated either with linezolid trough concentration (C_min_) [8,11,12,13,14,15,16,17] or an area under the plasma concentration time curve between 0 and 24 h post-dose (AUC_0–24h_) [15,18,19]. Studies by Song et al. [8] and Eimer et al. [16] suggest a target linezolid trough concentration of <2.0 mg/L for the prevention of toxicity. In the study by Song et al., which was conducted in 38 patients, all patients that exceeded the target suffered from adverse events [8]. However, Song et al. investigated only C_min_ versus toxicity and no other pharmacokinetic (PK) parameters such as AUC_0–24h_ as a driver for toxicity. In order to better understand and characterize the exposure–safety relationship between linezolid exposure and toxicity, a model-based approach was applied in this work to derive safety targets for linezolid-induced occurrence of anemia, peripheral neuropathy, thrombocytopenia and severe thrombocytopenia using time-to-event (TTE) and indirect response modelling approaches.

## 2. Results

### 2.1. Patients

In total, 75 patients were included in the analysis. Patient characteristics are presented in Table 1 and a summary of co-medications in Table S1. Patients suffering from the adverse event already at the start of linezolid treatment or that had missing information regarding the event were excluded from the analysis, resulting in 38, 73 and 50 patients included in the analysis for anemia, thrombocytopenia and peripheral neuropathy, respectively. Optic neuritis was excluded from the analysis since the incidence was low in this population, with only one patient (1%). In total, 58% of the patients suffered from anemia, 26% from peripheral neuropathy, 21% from thrombocytopenia and 1% from severe thrombocytopenia (Table 2). The median linezolid treatment length was 182 days (range: 16–611 days). Between the first and last dose, 63% of the patients had a dose reduction, 37% were kept on the same daily dose and for 0% the daily dose was increased. Dose reductions were executed in 79% of the patients receiving a starting dose of 1200 mg daily and in 57% receiving a starting dose of 600 mg daily. More details regarding dose changes between the start and end of treatment are shown in Figure S1.

### 2.2. Exposure–Safety Relationship

#### 2.2.1. C_min_ for Anemia and Peripheral Neuropathy

**Figure 1:**
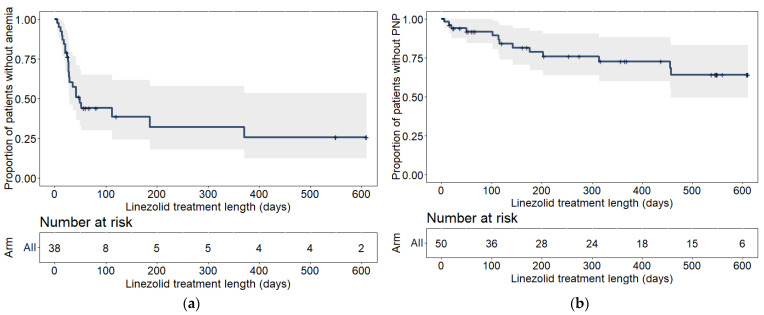
Kaplan–Meier plots for the time-to-event for (**a**) anemia and (**b**) peripheral neuropathy. The shaded area represents the standard error, computed using the Greenwood method [21]. Vertical dashes represent censoring, while downward steps represent events. The number at risk table represents the total number of participants without the event or censoring. PNP, peripheral neuropathy.

In total, 58% (22/38) of the participants had an anemia adverse event and 26% (13/50) of the participants developed peripheral neuropathy during linezolid treatment. Kaplan–Meier plots of the raw data are shown in Figure 1.

A Gompertz distribution best described the baseline hazard for both anemia and peripheral neuropathy. An exposure–response relationship between linezolid C_min_ and either adverse event was identified, which provided the best fit compared to the other tested exposure indices. The exposure–response relationship was in both cases best described by a power function. No patient characteristics were identified to be statistically significant predictors. The final parameter estimates can be found in Table 3. A graphical model evaluation using visual predictive checks (VPCs) showed that the models described the data well since the observed data lie within the 95% prediction intervals of the model predictions (Figure S2).

**Figure 2:**
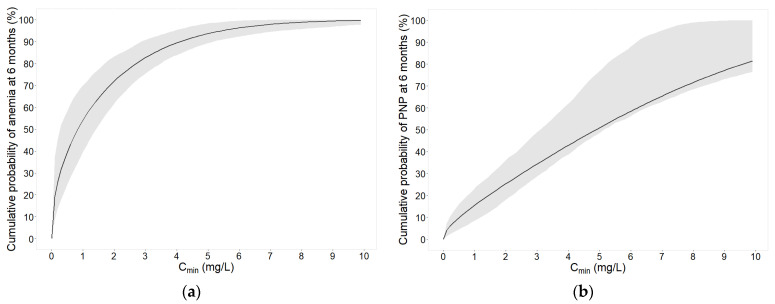
Probability of developing the event within 6 months of treatment (cumulative probability at 6 months) versus linezolid exposure in a typical linezolid patient for (**a**) anemia and (**b**) peripheral neuropathy (PNP). The solid line represents the median model prediction given the model parameters. The grey shaded area indicates the 95% confidence interval given the uncertainty in the model parameters (1000 samples).

The cumulative probabilities to develop anemia or peripheral neuropathy within 6 months versus linezolid C_min_ as well as versus time at different dose groups are displayed in Figure 2 and Figure S3, respectively.

#### 2.2.2. AUC_0–24h_ for Thrombocytopenia

**Figure 3:**
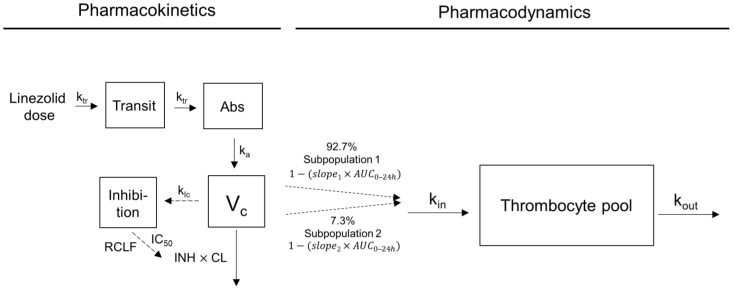
Schematic representation of the final pharmacokinetic–pharmacodynamic (PKPD) indirect response model used to describe the change in thrombocyte counts during linezolid treatment. The PK model has been developed previously [22]. Abs, absorption compartment; AUC_0–24h_, area under the linezolid plasma concentration time curve up to 24 h; CL, linezolid clearance; IC_50_, half of the maximum possible clearance inhibition; INH, concentration and time dependency of the inhibition; k_a_, absorption rate constant; k_Ic_, rate constant representing the transfer from the central into the inhibition compartment; k_in_, zero-order input rate; k_out_, first-order fractional turn-over rate; k_tr_, transit rate constant; RCLF, fraction of clearance remaining uninhibited; slope_1_, linear drug effect for patients belonging to subpopulation 1 (decreasing thrombocyte count); slope_2_, linear drug effect for patients belonging to subpopulation 2 (increasing thrombocyte count); V_c_, central linezolid compartment.

In total, 73 patients were included in the analysis, out of which 15 (21%) and 1 (1%) had thrombocytopenia and severe thrombocytopenia, respectively. A model where linezolid inhibits the formation of thrombocytes best described the data. The difference in OFV compared to a model where linezolid stimulates thrombocyte elimination was −5.9. The exposure index with the highest correlation to thrombocyte count was the AUC_0–24h_, implemented as a linear exposure–response relationship. Visual inspection of the raw data indicated that the thrombocyte count increased with linezolid treatment for a small proportion of patients. In order to facilitate both a decrease and an increase in thrombocyte counts over time, a mixture model was applied, allowing for a negative or positive slope. The implementation of the mixture model led to a drop in the objective function value (OFV) of 7.6 for 2 degrees of freedom, which is statistically significant on a 0.025 significance level. The proportion of patients belonging to the subpopulation with a decrease in thrombocyte count during linezolid treatment (subpopulation 1) was estimated to 92.7% (Table 4), indicating that linezolid treatment leads to a reduced thrombocyte count in the majority of the patients. In a small proportion of patients (7.3%), the thrombocyte count increased with treatment (subpopulation 2). The baseline thrombocyte count (*base*) was estimated to 279·10^9^/L, the thrombocyte production rate (*k_in_*) to 6.72·10^9^/L/day, the *slope* describing the drug effect for patients with decreasing thrombocyte counts to 0.0022 mg^−1^·L·h^−1^ and for patients with increasing thrombocyte counts to −0.0027 mg^−1^·L·h^−1^ (Table 4). The residual error model included a proportional error, which led to a lower OFV (−162.1) compared to an additive error model. A combined additive and proportional error model did not improve the fit. Inter-individual variability (IIV) in *base*, *k_in_*, *slope* and residual error was statistically significant. Having two different IIVs on *slope* for the two different subpopulations did not improve the model fit. Age was identified as a statistical covariate on *slope*. Graphical model evaluation using prediction-corrected VPC (Figure S4) and individual plots showed good model performance. Final parameter estimates are shown in Table 4 and the structure of the final model is illustrated in Figure 3.

**Figure 4:**
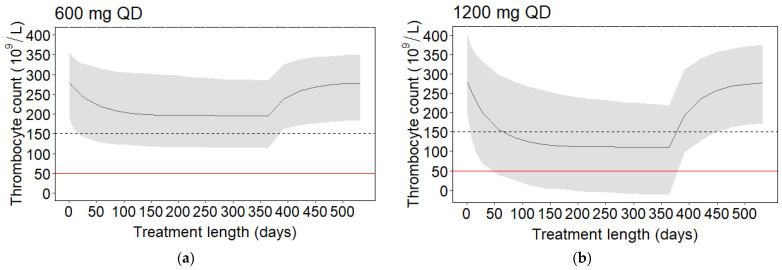
Simulated thrombocyte counts over time in the study population for 1 year of linezolid treatment based on the final model for a dose of (**a**) 600 mg QD and (**b**) 1200 mg QD. After 365 days, treatment is stopped and the thrombocyte recovery is shown. The black solid line represents the thrombocyte count for the typical individual. The black dashed line and the red solid line indicate thrombocytopenia (<150·10^9^/L) and severe thrombocytopenia (<50·10^9^/L), respectively. The shaded area is the 80th prediction interval of the variability within the population, including inter-individual variability (1000 individuals).

Simulations of thrombocyte counts versus time at different doses for patients with decreasing thrombocyte counts, i.e., subpopulation 1, showed that the thrombocyte count decreases with treatment but recovers after stopping treatment (Figure 4).

#### 2.2.3. Dropout

In total, 29/75 (39%) patients stopped linezolid treatment before 3 months. Out of these 29 patients, 22 (76%) experienced an adverse event, of which 62% had anemia, 24% thrombocytopenia and/or 14% peripheral neuropathy. Out of the patients that dropped out, 50% received a daily starting dose of 1200 mg and 50% received 600 mg. A graphical analysis of the raw data is presented in (Figure S5) as a Kaplan–Meier plot. The final dropout TTE model consisted of a baseline hazard following a log-normal distribution and a linear exposure–response relationship between linezolid C_min_ and dropout, where increasing C_min_ leads to increased probability of dropout (Figure S6b). Final model parameter estimates and VPCs can be found in Table 5 and Figure S6, respectively.

#### 2.2.4. Safety Targets and Simulations

A cumulative probability of 20% to develop either adverse event at 6 months of treatment was selected for the safety target determination. According to the model predictions, the safety targets were a C_min_ of <0.11 mg/L and <1.4 mg/L for anemia and peripheral neuropathy, and an AUC_0–24h_ of <111 h·mg/L and <270 h·mg/L for thrombocytopenia and severe thrombocytopenia, respectively. In addition, a summary of the safety targets for different treatment lengths is provided in Table 6. With increasing intended treatment lengths, the targeted linezolid exposure index needs to be lower (Table 6).

Furthermore, C_min_ and AUC_0–24h_ targets for the severe adverse events peripheral neuropathy and severe thrombocytopenia corresponding to cumulative probabilities below 20% are presented in Table S2.

At the currently recommended dose of 600 mg QD for 6 months [5], the model-predicted cumulative probability for anemia and peripheral neuropathy was 57.1% and 16.9%, respectively (Figure S3). In total, 21.6% and 1.2% of patients were predicted to develop thrombocytopenia and severe thrombocytopenia, respectively. At a higher dose of 1200 mg QD for 6 months, the model-predicted cumulative probability for anemia and peripheral neuropathy was 78.2% and 30.2%, respectively (Figure S3). In total, 56.0% and 19.7% of patients were predicted to develop thrombocytopenia and severe thrombocytopenia, respectively. At the lower dose of 300 mg QD for 6 months, the model-predicted cumulative probability for anemia and peripheral neuropathy was 40.3% and 10.1%, respectively (Figure S3). In total, 5.6% and 0% of patients were predicted to develop thrombocytopenia and severe thrombocytopenia, respectively. 

Model predictions showed that at the currently commonly used C_min_ target of 2.0 mg/L (total concentration), the cumulative probability at 6 months of treatment was 72.2% for anemia (Figure 2a) and 25.5% for peripheral neuropathy (Figure 2b), respectively.

#### 2.2.5. Clinical Utility Assessment

**Figure 5:**
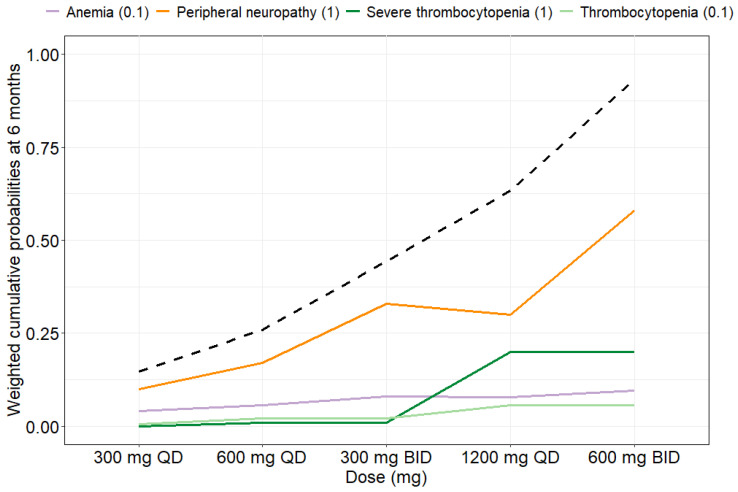
Weighted cumulative probabilities at 6 months (weighting) and clinical utility index (CUI) for the safety endpoints. The weights for peripheral neuropathy, anemia, thrombocytopenia and severe thrombocytopenia are 1:0.1:0.1:1. The black dashed line indicates the CUI derived as the sum of the weighted cumulative probabilities for all four endpoints. QD, once daily; BID, twice daily.

Clinical utility was assessed to compare the toxicity of different dosing regimens considering all four safety endpoints. The clinical utility index (CUI) was calculated using the final exposure–response models using a weighting of 1:0.1:0.1:1 for peripheral neuropathy, anemia, thrombocytopenia and severe thrombocytopenia, respectively. In Figure 5, the CUI, i.e., the sum of the weighted cumulative probabilities at 6 months, as well as the individual weighted cumulative probabilities for each endpoint, are given. Figure S7 contains the cumulative probabilities at 6 months without weighting for each endpoint. Based on the CUI assessment, it becomes obvious that a dose of 600 mg QD seems safe considering all four safety targets. A twice daily (BID) dosing strategy of 300 mg BID is worse from a safety perspective for anemia and peripheral neuropathy since those endpoints are driven by C_min_. A dose of 1200 mg QD leads to too high toxicity. A sensitivity analysis showing the clinical utility for different weights applied to the safety endpoints can be found in Figure S8.

## 3. Discussion

In this work we derived new safety targets for linezolid treatment in TB patients suggesting that at the currently recommended treatment length of 6 months, a C_min_ of 0.11 mg/L and 1.4 mg/L should not be exceeded to keep the cumulative probability to develop anemia and peripheral neuropathy below 20%, and the AUC_0–24h_ should be below 111 h·mg/L or 270 h·mg/L to keep the cumulative probability to develop thrombocytopenia and severe thrombocytopenia below 20%, respectively. At the currently recommended dose of 600 mg QD for 6 months [5], the model-predicted cumulative probability for anemia, peripheral neuropathy, thrombocytopenia and severe thrombocytopenia was 57.1%, 16.9%, 21.6% and 1.2%, respectively. A clinical utility assessment showed that a dose of 600 mg QD on the typical level seems safe considering all four safety endpoints. A BID dosing strategy of 300 mg BID is inferior from a safety perspective for anemia and peripheral neuropathy since those endpoints are driven by C_min_. A dose of 1200 mg QD leads to too high toxicity, as confirmed in the Nix-TB trial [2]. 

Establishing exposure–toxicity relationships and safety targets based on clinical data are crucial to enable safe dosing of linezolid considering its high toxicity. Safety targets enable model-informed precision dosing (MIPD) for individualized dosing, which aims to ensure safe and efficacious dosing in every patient [22,23]. Previous work highlights the importance of therapeutic drug monitoring (TDM) for linezolid [7,11,24,25,26,27]. Due to large IIV in linezolid PK and its small therapeutic window, an individualized dosing strategy would likely be beneficial for the patients. MIPD could be applied using the here presented newly proposed safety targets and the already established efficacy target of free AUC_0–24h_/MIC (*f*AUC_0–24h_/MIC) > 119, corresponding to a total AUC_0–24h_/MIC > 173 [6,28], to optimize the dose on an individual level. The final dose is thus also dependent on the bacterial susceptibility, i.e., MIC, highlighting the importance of MIC determination. The dose can be lowered in case of a lower MIC, which also contributes to increased safety.

In this real world TDM patient population, the incidence of linezolid-induced anemia was 58% (Table 2). For comparison, in a French study performed by Eimer et al. [16], the occurrence of myelosuppression was 11%, i.e., lower than in the here described patient population. However, the authors defined myelosuppression as severe myelosuppression, whilst in the here described work, anemia and not severe anemia was used as an endpoint. The incidence of severe anemia was 1% (Table 2), which is more comparable to the results by Eimer et al. A study conducted in a South African population by Wasserman et al. [17] reported similar incidences for anemia (38%). In our work, a relationship between linezolid C_min_ and anemia was identified where increasing trough concentrations increased the risk of developing anemia (*p* < 0.01). No other covariates were found to be statistically significant. Even though some studies suggest that renal function can impact linezolid toxicity [11,12], creatinine clearance was not identified as a risk factor to develop anemia (or peripheral neuropathy and thrombocytopenia) in this work. This could be due to the fact that only two patients in this population had mild renal impairment with a creatinine clearance between 50 and 80 mL/min (calculated based on the Cockcroft–Gault formula [20]), whilst all other patients exhibited normal kidney function. Therefore, it might not have been possible to detect the impact of renal impairment on linezolid toxicity in this study population. Based on the final anemia pharmacokinetic–pharmacodynamic (PKPD) model, a safety target was determined. A 6 month treatment length was selected for target determination since the results of the ZeNix trial indicate a positive outcome in regards to efficacy and safety at 6 months of treatment [3], and these findings were recently adopted into WHO guidelines [5]. Based on a 20% cumulative probability to develop anemia within 6 months of treatment, the here derived safety target was a C_min_ below 0.11 mg/L. This target C_min_ might be considered low and the risk for anemia should be balanced against efficacy. Anemia may be tolerable in the clinic when necessary to ensure efficacy, while severe anemia is a critical condition that should be avoided. Since severe anemia is of higher clinical relevance than anemia, there is an unmet need to identify a safety target for severe anemia, which could not be investigated here due to only one patient suffering from severe anemia. At the commonly applied C_min_ safety target of <2.0 mg/L, the model-predicted cumulative probability at 6 months was 72.2% (cumulative probability at 1 year: 82.4%) for anemia (Figure 2a).

The occurrence of linezolid-induced peripheral neuropathy was 26% (Table 2). This is very similar to the incidence reported previously by Cui et al. [29], Eimer et al. [16] and Wasserman et al. [17], who reported occurrences of 28%, 34% and 25%, respectively. A statistically significant nonlinear relationship between linezolid C_min_ and peripheral neuropathy was identified using a TTE approach where increasing trough concentrations increased the risk of developing the adverse event (*p* < 0.01). No other covariates were found to be statistically significant. The here derived safety target was a C_min_ below 1.4 mg/L. At the commonly applied C_min_ safety target of <2.0 mg/L, the model-predicted cumulative probability at 6 months was 25.5% (cumulative probability at 1 year: 41.5%) (Figure 2b).

The incidence of linezolid-induced thrombocytopenia and severe thrombocytopenia was 21% and 1%, respectively (Table 2), which is comparable to the occurrence of thrombocytopenia in the study conducted by Wasserman et al. (7%) [17]. In order to investigate the exposure–response relationship for thrombocytopenia and severe thrombocytopenia, an indirect response model describing thrombocyte counts over time was developed. A model where linezolid inhibits the thrombocyte production described the data slightly better than a model where linezolid stimulates the thrombocyte elimination. However, conclusions regarding which mechanism is more likely should not be drawn based on this work. This should be investigated in further studies. The exposure–response relationship was described as a linear relationship between the AUC_0–24h_ and production of thrombocytes. In the majority of the patients (92.7%) (Table 4), the thrombocyte counts decreased with linezolid treatment length and AUC_0–24h_, while 7.3% of the patients were classified as having an increase in thrombocyte counts with treatment. The increase in thrombocyte counts despite treatment could be explained by an overall improvement of the patient’s health status. Age was found to be a statistically significant covariate on the drug’s effect on the thrombocyte production rate. The effect of age on *slope* was 1.02, meaning that an older patient has a larger decrease in thrombocyte counts during linezolid treatment than a younger patient. Monte Carlo simulations from the final thrombocyte indirect response model were performed to predict the linezolid AUC_0–24h_ resulting in (severe) thrombocytopenia in 20% of the patients at different treatment lengths (Table 6). The derived safety targets were an AUC_0–24h_ of <111 h·mg/L and <270 h·mg/L for thrombocytopenia and severe thrombocytopenia, respectively. Simulations of thrombocyte counts during and after treatment in the typical individual showed that after 1 year of treatment, the thrombocyte counts recover within 6 months irrespective of treatment with 600 mg or 1200 mg QD (Figure 4). 

In order to compare the toxicity of different dosing regimens, a clinical utility approach [30,31] was applied where the four different endpoints were weighted based on their severity (Figure 5 and Figure S8). The CUI offers support to clinicians to compare the safety of different dosing regimens. Here, it is important to keep in mind that the CUI value itself should only be considered when comparing dosing regimens from the safety perspective, and the value outside of this context is of no meaning. Since in this work the CUI is applied to weight safety endpoints against each other, a higher CUI means a higher toxicity. The CUI concept may also be applied for MIPD of linezolid using the here developed safety PKPD models to compute the patient’s individual CUI. The weighting of the different safety endpoints can also be individualized based on each specific patient case. Evaluation of clinical utilities should consider relevant clinical weights of probabilities of the different safety endpoints. As peripheral neuropathy can be irreversible and severe thrombocytopenia, even though reversible, can be life-threatening and needs transfusion before many (medical) interventions, these were given more weight. Anemia and thrombocytopenia are reversible but can be very well tolerated and thus were weighted much less. The exact weighting is, however, open for debate. Since the weighting impacts the CUI and subsequently the conclusions drawn regarding a tolerated dose, we performed a sensitivity analysis exploring different weights (Figure S8). These additional scenarios can be used to see what the CUI would be given different weights as clinicians might want to apply different weights to prioritize the prevention of adverse events differently. The clinical utility assessment showed that 600 mg QD seems safe on the typical level, which supports the usage of the WHO’s currently recommended dose [5]. This dose has been shown to be both efficacious as well as safe in the ZeNix trial, where 91% of participants had a favorable outcome and a substantial decrease in adverse events was observed [3] compared to the Nix-TB trial [2] where a dose of 1200 mg QD was investigated. From the results presented in Figure 5 and Figures S7 and S8, it becomes evident that a BID dosing is worse from a safety perspective for the safety endpoints that are related to C_min_, i.e., peripheral neuropathy and anemia. This highlights the importance of a QD dosing strategy for linezolid. Since efficacy has been shown to be driven by the *f*AUC_0–24h_ [6,28], the decision on using a QD versus BID approach should not impact the efficacy. The superiority of a QD dosing over BID has been demonstrated previously [6,22,28,32] and is also beneficial to improve patient adherence.

Both C_min_ and AUC_0–24h_ were identified as exposure indices driving either peripheral neuropathy and anemia or thrombocytopenia. This might be due to the different modelling approaches used for peripheral neuropathy and anemia versus thrombocytopenia. Another reason could be that linezolid C_min_ and AUC_0–24h_ have been shown to be correlated [11,17,28,33]. As mentioned above, C_min_ has previously been associated with mitochondrial linezolid toxicity [8,16,17], supporting a QD rather than BID dosing strategy, but also the AUC_0–24h_ has been suggested to be related to toxicity [15,18,19].

In total, 39% patients dropped out, i.e., stopped treatment before 3 months. The probability of dropping out was found to be related to linezolid C_min_ (Figure S6b). At doses of 600 mg QD, 300 mg BID and 1200 mg QD, the typical probabilities to drop out within 3 months were 9%, 45% and 40%, respectively. In this analysis, the dropout was considered as missing at random (MAR), i.e., dependent on the observed value of the dependent variable [34,35,36,37], which in this case was linezolid exposure. Dropout that is MAR should be taken into account when performing simulations [34], which was done for the thrombocyte count Monte Carlo simulations. 

A limitation of this work is that the here analyzed adverse events are assumed to be caused by linezolid and not by any of the other drugs in the individual background regimen. However, linezolid is known to cause myelosuppression and peripheral neuropathy [38], while the other drugs, including bedaquiline and fluoroquinolones, do not commonly cause these adverse events. In addition, performing a study where linezolid is administered in monotherapy over a prolonged time period would ethically not be justifiable. Another limitation of this work is the fact that only time-to-anemia was modeled, while time-to-severe anemia (defined as <5.0 g/dL) could not be determined because only one patient in the patient population suffered from severe anemia. Furthermore, the safety target for severe thrombocytopenia should be interpreted with caution since only one patient in the analysis data set experienced severe thrombocytopenia and thus the model predictions from the thrombocyte count indirect response model are extrapolated and may be uncertain. The difference in handling the fact that only one patient experienced severe anemia and severe thrombocytopenia lies in the nature of the two modelling approaches. For anemia, a TTE approach was applied. Developing a time-to-severe anemia model based on one patient is not possible. For thrombocytopenia, however, we applied a continuous indirect response model where the thrombocyte count over time was modelled and not thrombocytopenia or severe thrombocytopenia per se. From this continuous model, thrombocyte counts at different timepoints and linezolid exposures can be simulated and the proportion of simulated patients falling below the thresholds for thrombocytopenia or severe thrombocytopenia can be derived. In addition, it was not possible to develop a model describing optic neuritis because only one patient experienced this adverse event. Lastly, the fact that TDM data were used where some patients’ doses were adjusted due to adverse events may have impacted the estimation of the exposure–response relationship. 

## 4. Materials and Methods

### 4.1. Patients and Data

TDM data from 75 MDR-TB or XDR-TB patients treated with linezolid for up to 20 months at the TB center Beatrixoord in Haren, University Medical Center Groningen, The Netherlands (2007–2021), were analyzed. Since the study was performed retrospectively and because TDM was already a part of the routine treatment protocol in the hospital, a waiver for informed consent from the patients was issued by the Medical Ethical Review Board UMCG (METC 2013.492). The collected data included patient demographics and characteristics, total (unbound and bound) linezolid plasma concentrations, linezolid dosing information, as well as safety data including the occurrence of peripheral neuropathy, optic neuritis and anemia during treatment, and thrombocyte counts during and after linezolid treatment. Patients were treated with linezolid in combination with other drugs commonly used in TB therapy, with linezolid doses ranging from 150 mg to 1200 mg daily. An overview of the administered co-medications is provided in Table S1. Plasma linezolid concentrations were quantified using liquid chromatography coupled with mass spectrometry (LC-MS/MS) with a lower limit of quantification of 0.05 mg/L [39]. Patients with missing information on the respective safety endpoint were excluded from the analysis of that endpoint.

### 4.2. Pharmacokinetic Data and Exposure Indices

In order to determine the exposure–response relationship between linezolid plasma concentrations and the four safety outcomes of anemia, peripheral neuropathy, thrombocytopenia and severe thrombocytopenia, individual linezolid exposure was derived based on individual Bayes estimates (EBEs) obtained using a previously developed linezolid population PK model [22], individual observed plasma concentrations and covariate information. As time-varying exposure indices, the individual AUC_0–24h_, average plasma concentration (C_av_) (calculated as model-predicted AUC_0–24h_/dosing interval), maximal concentration within a dosing interval (C_max_) and C_min_ were predicted from the linezolid population PK model [22] matching the day of the adverse event observation. This means that the predicted individual exposure indices varied with treatment time and potential changes in dosing. Wherever covariate information was missing, it was replaced by the population median for continuous covariates and the mode for categorical covariates. In cases of missing PK information (*n* = 5), the typical patient exposure for the respective dose group was used, accounting for covariates.

### 4.3. Exposure–Response Analysis

#### 4.3.1. TTE Modelling

The peripheral neuropathy data were a time-to-first event type of data. For anemia, level data were reported where the severity of anemia was described on a scale consisting of six grades. Anemia was defined as a hemoglobin count <8.5 g/dL and severe anemia as a hemoglobin count <5.0 g/dL according to CTCAE [40]. However, because only one patient suffered from severe anemia, all anemia data were treated as time-to-first anemia event without categories. No information about repeated peripheral neuropathy or anemia was available. Event times were calculated as days since the start of linezolid treatment to adverse event. Patients were censored one day after stopping linezolid treatment or at the day of last assessment. Two separate TTE models were developed: one for peripheral neuropathy and one for anemia.

For each of the TTE models, an exponential (Equation (1)), a Weibull (Equation (2)), a Gompertz (Equation (3)) and a log-normal (Equation (4)) distribution were explored as baseline hazard functions. After determining the TTE baseline hazard function, patient covariates were investigated as risk factors. Tested covariates included age, sex, bodyweight, WHO region of origin, presence of diabetes mellitus, smoking status, creatinine clearance, disease-related malnutrition and pre-emptive use of erythropoietin. Creatinine clearance was calculated from serum creatinine values using the Cockcroft–Gault formula [20] and was truncated to 150 mL/min due to scientific plausibility. Pregnancy, HIV and alcohol abuse were excluded from the covariate analysis due to scarcity of data (5%, 7% and 8% respectively). Covariates were included in a forward selection step at a *p* ≤ 0.05 significance level and retained if still statistically significant after backwards deletion (*p ≤* 0.01). Finally, the different time-varying linezolid pharmacokinetic exposure indices (AUC_0–24h_, C_av_, C_max_ and C_min_) were evaluated as risk factors for developing peripheral neuropathy or anemia. Linear, exponential, power, E_max_ and sigmoidal E_max_ functions were investigated to describe the exposure–response relationship.

h_0_(*t*) = *λ* (1)

h_0_(*t*) = *λγ*(*λt*)*^γ^*^−1^ (2)

h_0_(*t*) = *λe^γt^* (3)

h_0_(*t*) = *f*(*t*)*/F*(*t*) (4)

In Equation (4), *F*(*t*) (cumulative distribution function) and *f*(*t*) (probability distribution function) are defined as:

$$F\left(t\right)=\Phi \;\left(\frac{\log\left(t\right)-\mu }{\sigma }\right)$$

$$f\left(t\right)=\frac{\Phi \left(\frac{\log\left(t\right)-\mu }{\sigma }\right)}{t\sigma }$$

The parameters *λ* and *γ* are the scale and shape factors of the Weibull and Gompertz distribution, respectively. The parameters μ and σ are the mean and standard deviation of the log-normal distribution, respectively. The parameter *Φ* is the cumulative distribution function of the normal distribution and *h*_0_(*t*) is the baseline hazard. The hazard was modelled as described in Equation (5).

*h*(*t*) = *h*_0_(*t*) · *e*^f(C(*t*)) + *β*_1_·*X*_1_ + … + *β*_n_·*X*_n_^ (5)

In Equation 5, *h*(*t*) represents the hazard of developing the respective adverse event over time, *h_0_(t)* the baseline hazard (from Equations (1)–(4)), *f*(*C*(*t*)) the exposure–response relationship and *β_n_* the effect of a risk factor *X_n_.* The covariate effects were parameterized as [1 + *β_n_*·(*X_n_* − *X_n_*_,*median*_)] for continuous covariates and as *β_n_* · *X_n_* for categorical covariates. Due to fact that information on the respective adverse events was only captured at the start and during treatment, it was not possible to estimate the baseline hazard to develop anemia or peripheral neuropathy without linezolid treatment. Thus, the baseline hazard was parameterized as being dependent on the linezolid exposure, i.e., when drug exposure is 0 mg/L, the hazard is 0. This means that the hazard *h*(*t*) describes the hazard to develop the respective adverse event as a cause of linezolid exposure in addition to the baseline risk without treatment.

#### 4.3.2. Indirect Response Modelling

Due to the continuous nature of the thrombocyte count data, thrombocyte counts over time were modelled using an indirect response modelling approach. Thrombocytopenia (defined as thrombocyte count <150·10^9^/L according to the CTCAE [40]) and severe thrombocytopenia (defined as thrombocyte count <50·10^9^/L according to the CTCAE [40]) were derived from the indirect response model. Baseline individual thrombocyte counts were set to the pre-treatment observation closest to the start of linezolid treatment. Thrombocyte counts in 10^9^/L were modelled over time during linezolid treatment as well as after stopping treatment in order to also characterize the thrombocyte recovery after stopping treatment. Previous works indicate that linezolid either inhibits the formation of thrombocytes [41,42,43] or stimulates their elimination [10,44,45]. Since no consensus has been reached as to which mechanism is more likely, both the inhibition of thrombocyte formation by linezolid (Equation (6)) and stimulation of elimination (Equation (7)) were evaluated as possible mechanisms of action.

(6) $$\frac{dPt}{dt}=k_{in}\cdot \left(1-\left(Slope\cdot C\left(t\right)\right)\right)-k_{out}\cdot Pt$$

(7) $$\frac{dPt}{dt}=k_{in}-k_{out}\cdot \left(1+\left(Slope\cdot C\left(t\right)\right)\right)\cdot Pt$$

Equations (6) and (7) describe the thrombocyte count *Pt* over time, where *k_in_* is the thrombocyte formation rate, *k_out_* the thrombocyte elimination rate, *slope* the drug effect on thrombocyte production/elimination and *C*(*t*) the time-varying linezolid exposure index (AUC_0–24h_, C_av_, C_max_ or C_min_). In addition to a slope function, power, E_max_ and sigmoidal E_max_ relationships were tested to describe the exposure–response relationship. The parameters *k_in_* and the baseline thrombocyte count *base* were estimated and *k_out_* calculated as *k_out_* = *k_in_/base*. This parameterization assumes that the formation and elimination of thrombocytes is at a steady state before treatment start and that disease progression does not impact the thrombocyte count.

A graphical analysis of the raw data revealed that the thrombocyte counts increased in a few patients during treatment. To facilitate an increase in thrombocyte count over time in some patients, the implementation of either an additive IIV on *slope* or a mixture model were tested. For the mixture model, the $MIXTURE subroutine in NONMEM [46] was used which assigns individual patients to a subpopulation, where the probability for a patient belonging to a subpopulation and finally the fraction of patients in either subpopulation are estimated [47]. The mixture feature was tested on both fixed and random effects describing the drug’s effect on thrombocyte production rate over time, including: (1) two different *slope* estimates per subpopulation, (2) either a positive or negative *slope* per subpopulation with the same *slope* magnitude and (3) different IIV estimates for *slope* per subpopulation. 

Covariates of age, sex, bodyweight, WHO region of origin, diabetes mellitus, smoking status, creatinine clearance, disease-related malnutrition and pre-emptive use of erythropoietin were evaluated to influence the drug’s effect, implemented as linear, power or exponential relationships. Covariates were included in a forward selection step at a *p ≤* 0.05 significance level and retained if still statistically significant after backwards deletion (*p ≤* 0.01). IIV was tested on *k_in_, base, slope* and the residual error. During graphical analysis, a large variability in the data was visible, and thus IIV on residual error was tested to evaluate variability between patients in their residual error after testing all other structural, stochastic and covariate components. Different residual error models including additive, proportional and combined error models were tested.

### 4.4. Dropout Model

In order to account for dropout, which was assumed to be missing at random (MAR), i.e., dependent on linezolid exposure, a TTE model describing the time to dropout was built. Dropout was defined as stopping treatment <3 months. An exponential (Equation (1)), a Weibull (Equation (2)), a Gompertz (Equation (3)) and a log-normal (Equation (4)) distribution were explored as baseline hazard functions. After determining the TTE baseline hazard distribution, patient covariates were investigated as risk factors. Tested covariates included age, sex, bodyweight, WHO region of origin, diabetes mellitus, smoking status, creatinine clearance, disease-related malnutrition and pre-emptive use of erythropoietin. Next, different time-varying linezolid pharmacokinetic exposure indices (AUC_0–24h_, C_av_, C_max_ and C_min_) were tested as risk factors to drop out. Linear, exponential, power, E_max_ and sigmoidal E_max_ functions were evaluated to describe the exposure–response relationship.

### 4.5. Model Evaluation

The model evaluation was guided by the drop in OFV, the precision in parameter estimates, the magnitude of the condition number, minimization status, scientific plausibility and graphical model evaluation using VPCs and goodness of fit plots. The OFV, which approximates the −2log (likelihood) of the data, was utilized for the comparison of nested models using the likelihood ratio test. The difference between OFVs is approximately χ^2^-distributed, i.e., a drop of 3.84 in OFV would be statistically significant for one degree of freedom and a significance level of α = 0.05. Uncertainty in parameter estimates were reported as a 90% confidence interval derived from a nonparametric bootstrap (1000 samples).

### 4.6. Safety Target Derivation

In order to derive new clinical safety targets for anemia and peripheral neuropathy, the exposures corresponding to a cumulative probability of 20% to develop either adverse event within 6, 9 or 12 months of treatment were derived. Dropout was not taken into account in the calculation of the cumulative probability. The safety target was based on the currently recommended treatment length of 6 months [5]. 

Safety targets for thrombocytopenia and severe thrombocytopenia were derived by performing Monte Carlo simulations for 1000 virtual patients including IIV from the final indirect response model to identify the exposure index values where the thrombocyte counts in 80% (corresponding to a cumulative probability of 20%) of the patients do not drop below 150·10^9^/L (thrombocytopenia) and 50·10^9^/L (severe thrombocytopenia) within 6, 9 or 12 months of treatment. In the simulations, the mixture parameter was fixed to 0, i.e., simulating only patients with a decrease in thrombocyte count. Dropout was taken into account in the simulations. The safety target was based on a treatment length of 6 months. 

Using a cumulative probability of 20% as a safety target was deemed to be acceptable after discussions with clinicians and hospital pharmacists. However, to provide further information for clinical judgement, the exposures corresponding to cumulative probabilities of 5% and 10% were derived in addition for the severe adverse events of peripheral neuropathy and severe thrombocytopenia.

### 4.7. Clinical Utility Index

In order to compare the safety of different dosing regimens considering all four safety targets, the CUI was calculated as

$$CUI=\overset{n}{\underset{i=1}{\sum }}w_{i}\cdot p_{endpoint,i}\left(event\leq T\right)$$

where *n* is the number of endpoints, *w_i_* the weight for endpoint *i* and *p_i_*(…) the event probability up to time T (i.e., the cumulative probability at time T) for corresponding endpoint *i*. The safety endpoints used in the clinical utility assessment were peripheral neuropathy, anemia, thrombocytopenia and severe thrombocytopenia. A weighting ratio of 1:0.1:0.1:1 was applied and cumulative probabilities were calculated at 6 months of treatment, indicating that peripheral neuropathy and severe thrombocytopenia were weighted more than the other two adverse events. The CUI was investigated over a range of dosing regimens. A sensitivity analysis exploring different weighting ratios was performed. 

### 4.8. Software

Model building as well as Monte Carlo simulations were performed in NONMEM 7.4.3 (Icon Development Solutions, Hanover, MD, USA) [46] together with PsN 5.0.0 (Department of Pharmaceutical Biosciences, Uppsala University, Uppsala, Sweden) [48]. Parameter estimation in the TTE modelling was performed using the likelihood estimation method, and the first order conditional estimation method with interaction (FOCE-I) was used for the indirect response modelling. Dataset preparation, data visualization, graphical analysis and model evaluation were performed in R statistical software version 4.0.3 (R Foundation for Statistical Computing, Vienna, Austria) [49].

## 5. Conclusions

In this work, we showed that increasing linezolid exposure increased the probability to develop anemia, peripheral neuropathy and (severe) thrombocytopenia. New safety targets for linezolid treatment in TB patients were derived, which were a C_min_ of <0.11 mg/L and <1.4 mg/L for anemia and peripheral neuropathy, and an AUC_0–24h_ of <111 h·mg/L and <270 h·mg/L for thrombocytopenia and severe thrombocytopenia, respectively. The newly proposed safety targets in combination with efficacy targets enable linezolid MIPD in drug-resistant tuberculosis patients, ensuring safe and efficacious dosing in every patient. The CUI may be utilized for MIPD using the here developed safety PKPD models to derive a patient’s individual CUI. A clinical utility assessment showed that a dose of 600 mg QD seems safe and superior compared to 300 mg BID on the typical level considering all four safety endpoints, which is in accordance with the WHO currently recommended dose. 

## Figures and Tables

**Table 1:** Patient characteristics included in the dataset used for pharmacokinetic–pharmacodynamic (PKPD) modelling.

Parameter	Unit	All Patients
N		75
Mean weight (range)	kg	60.7 (35.3–88.9)
Mean height (range)	m^2^	1.69 (1.50–1.93)
Mean creatinine clearance (range)	(mL/min)	116.1 (40.7–150.0) ^a,b^
Mean age (range)	years	32 (15–70)
Mean body mass index (range)	kg/m^2^	21.2 (15.5–32.6)
No. of male sex	n (%)	42 (56)
No. with HIV	n (%)	5 (7)
No. with Diabetes	n (%)	9 (12)
No. Smoking	n (%)	28 (38)
No. Alcohol abuse	n (%)	6 (8)
No. Pregnancy	n (%)	4 (5)
No. from indicated WHO region	n (%)	
African Region		11 (15)
Region of the Americas		2 (3)
South-East Asia Region		6 (8)
European Region		28 (37)
Eastern Mediterranean Region		17 (23)
Western Pacific Region		11 (15)

Age, bodyweight, body mass index and creatinine plasma concentration were registered on day of admission. WHO, WHO region classification describing origin of birth; alcohol, alcohol abuse characterized by more than 1 or 2 glasses of alcohol/day and less than 2 days/week with no alcohol. ^a^ Calculated using the Cockcroft–Gault equation [20], using lean body weight instead of regular body weight for patients with BMI higher than 25. ^b^ in 13/75 patients, creatinine clearance was truncated to 150 mL/min.

**Table 2:** Observed events related to safety endpoints.

Safety Endpoint	No. of Excluded Patients Due to Missing Information	No. of Excluded Patients Due to Adverse Event at Start of Treatment	Patients at Risk	No. of First Events (%)
Anemia ^a^	0	37	38	22 (58)
Severe anemia ^b^	0	0	75	1 (1)
Thrombocytopenia ^c^	0	2	73	15 (21)
Severe thrombocytopenia ^d^	0	2	73	1 (1)
Peripheral neuropathy	25	0	50	13 (26)
Optic neuritis	0	0	75	1 (1)

^a^ Anemia was defined as hemoglobin count <8.5 g/dL. ^b^ Severe anemia was defined as hemoglobin count <5.0 g/dL. ^c^ Thrombocytopenia was defined as platelet count <150·10^9^/L. ^d^ Severe thrombocytopenia was defined as platelet count <50·10^9^/L.

**Table 3:** Final exposure–response models describing the adverse events anemia and peripheral neuropathy.

		Anemia	Peripheral Neuropathy
Parameter	Description	Estimate (90% CI) ^a^	Estimate (90% CI) ^a^
*λ* (day^−1^)	Scale factor of the Gompertz distribution	0.004 (0.002–0.009)	0.0006 (0.0002–0.0013)
*γ*	Shape factor of the Gompertz distribution	−0.0056 (−0.015–−0.001)	−0.0011 (−0.0060–0.0032)
*β_ER_*·(C_min_) [(mg/L)^−1^]	Coefficient describing the exposure–response relationship as risk factor	0.417 (0.258–0.631)	0.464 (0.355–0.751)

CI, confidence interval; C_min_, linezolid trough concentration. ^a^ 90% CI is the 90% percentile confidence interval from a nonparametric bootstrap (1000 samples).

**Table 4:** Final parameter estimates for the thrombocyte indirect response model.

Parameter	Description	Typical Value (90% CI) ^a^	IIV ^b^ (90% CI) ^a^
*k_in_* (10^9^/L/day)	Zero-order input rate	6.72 (0.60–12.83)	1.82 (0.86–2.78)
*base* (10^9^/L)	Baseline thrombocyte count	279 (261.87–296.14)	0.0789 (0.05–0.11)
*Slope_1_* (mg^−1^·L·h^−1^)	Linear drug effect for patients belonging to subpopulation 1	0.0022 (0.001–0.003)	0.18 (0.04–0.32)
*Slope_2_* (mg^−1^·L·h^−1^)	Linear drug effect for patients belonging to subpopulation 2	−0.0027 (−0.004–−0.001)	0.18 (0.04–0.32)
*β_Age_*	Age effect on slope	0.0206 (0–0.048)	-
*p*	Proportion of patients belonging to subpopulation 2	0.073 (0–0.17)	-
*ϵ* (%)	Proportional residual error	2.07 (1.58–2.57)	0.103 (0.05–0.15)

Subpopulation 1 is the patient population with a decrease in thrombocyte count. Subpopulation 2 is the patient population with an increase in thrombocyte count. *p*, proportion of patients belonging to subpopulation 2 (1 − *p* is the proportion of patients belonging to subpopulation 1). ^a^ 90% CI is the 90% percentile confidence interval from a nonparametric bootstrap (1000 samples). ^b^ Inter-individual variability expressed as coefficient of variation.

**Table 5:** Final parameter estimates for the dropout time-to-event model.

Parameter	Description	Estimate (90% CI) ^a^
*µ*	Mean of the log-normal hazard model for dropout	5.22 (4.83–5.60)
*σ*	SD of the log-normal hazard model for dropout	0.983 (0.786–1.180)
*β_ER_* (C_min_) [(mg/L)^−1^]	Coefficient describing the exposure–response relationship as risk factor	0.251 (0.119–0.383)

CI, confidence interval; C_min_, linezolid trough concentration. ^a^ 90% CI is the 90% percentile confidence interval from a nonparametric bootstrap (1000 samples).

**Table 6:** Safety targets for different intended treatment lengths.

Treatment Length	Anemia	Peripheral Neuropathy	Thrombocytopenia	Severe Thrombocytopenia
	Target C_min_ (mg/L)	Target C_min_ (mg/L)	Target AUC_0–24h_ (h·mg/L)	Target AUC_0–24h_ (h·mg/L)
6 months	<0.11	<1.4	<111	<270
9 months	<0.08	<1.0	<110	<270
12 months	<0.06	<0.7	<106	<255

AUC_0–24h_, area under the linezolid plasma concentration time curve up to 24 h; C_min_, linezolid trough concentration.

## Data Availability

The data presented in this study are available on request from the corresponding author. The data are not publicly available due to privacy reasons, as these are sensitive personal patient data.

## Supplementary Materials

The following supporting information can be downloaded at: https://www.mdpi.com/article/10.3390/ph16111575/s1, Figure S1: Sankey plot showing individual dose adjustments; Figure S2: Kaplan–Meier Visual Predictive Check of the final linezolid exposure–response model for anemia and peripheral neuropathy; Figure S3: Cumulative probability of an event at different linezolid exposures versus treatment length for anemia and peripheral neuropathy; Figure S4: Prediction-corrected visual predictive check for the final thrombocyte indirect response model; Figure S5: Kaplan–Meier plot for the time to dropout event; Figure S6: Kaplan–Meier visual predictive check of the final dropout model showing dropout versus time and versus linezolid exposure; Figure S7: Model-predicted typical cumulative probabilities at 6 months for the four safety endpoints; Figure S8: Sensitivity analysis for the weighting applied to the clinical utility assessment; Table S1: Summary of co-medications; Table S2: Targets for peripheral neuropathy and severe thrombocytopenia for different target cumulative probabilities.

## References

[1] Global Tuberculosis Report 2022. https://www.who.int/teams/global-tuberculosis-programme/tb-reports/global-tuberculosis-report-2022.

[2] Conradie F., Diacon A.H., Ngubane N., Howell P., Everitt D., Crook A.M., Mendel C.M., Egizi E., Moreira J., Timm J. (2020). Treatment of Highly Drug-Resistant Pulmonary Tuberculosis. N. Engl. J. Med..

[3] Conradie F., Bagdasaryan T.R., Borisov S., Howell P., Mikiashvili L., Ngubane N., Samoilova A., Skornykova S., Tudor E., Variava E. (2022). Bedaquiline–Pretomanid–Linezolid Regimens for Drug-Resistant Tuberculosis. N. Engl. J. Med..

[4] Nyang’wa B.-T., Berry C., Kazounis E., Motta I., Parpieva N., Tigay Z., Solodovnikova V., Liverko I., Moodliar R., Dodd M. (2022). A 24-Week, All-Oral Regimen for Rifampin-Resistant Tuberculosis. N. Engl. J. Med..

[5] WHO Consolidated Guidelines on Tuberculosis. Module 4: Treatment—Drug-Resistant Tuberculosis Treatment, 2022 Update. https://www.who.int/publications-detail-redirect/9789240063129.

[6] Millard J., Pertinez H., Bonnett L., Hodel E.M., Dartois V., Johnson J.L., Caws M., Tiberi S., Bolhuis M., Alffenaar J.-W.C. (2018). Linezolid Pharmacokinetics in MDR-TB: A Systematic Review, Meta-Analysis and Monte Carlo Simulation. J. Antimicrob. Chemother..

[7] Rao G.G., Konicki R., Cattaneo D., Alffenaar J.-W., Marriott D.J.E., Neely M. (2020). Therapeutic Drug Monitoring Can Improve Linezolid Dosing Regimens in Current Clinical Practice: A Review of Linezolid Pharmacokinetics and Pharmacodynamics. Ther. Drug Monitor..

[8] Song T., Lee M., Jeon H.-S., Park Y., Dodd L.E., Dartois V., Follman D., Wang J., Cai Y., Goldfeder L.C. (2015). Linezolid Trough Concentrations Correlate with Mitochondrial Toxicity-Related Adverse Events in the Treatment of Chronic Extensively Drug-Resistant Tuberculosis. EBioMedicine.

[9] Leach K.L., Swaney S.M., Colca J.R., McDonald W.G., Blinn J.R., Thomasco L.M., Gadwood R.C., Shinabarger D., Xiong L., Mankin A.S. (2007). The Site of Action of Oxazolidinone Antibiotics in Living Bacteria and in Human Mitochondria. Mol. Cell.

[10] De Vriese A.S., Van Coster R., Smet J., Seneca S., Lovering A., Van Haute L.L., Vanopdenbosch L.J., Martin J.-J., Ceuterick-de Groote C., Vandecasteele S. (2006). Linezolid-Induced Inhibition of Mitochondrial Protein Synthesis. Clin. Infect. Dis..

[11] Pea F., Viale P., Cojutti P., Del Pin B., Zamparini E., Furlanut M. (2012). Therapeutic Drug Monitoring May Improve Safety Outcomes of Long-Term Treatment with Linezolid in Adult Patients. J. Antimicrob. Chemother..

[12] Nukui Y., Hatakeyama S., Okamoto K., Yamamoto T., Hisaka A., Suzuki H., Yata N., Yotsuyanagi H., Moriya K. (2013). High Plasma Linezolid Concentration and Impaired Renal Function Affect Development of Linezolid-Induced Thrombocytopenia. J. Antimicrob. Chemother..

[13] Matsumoto K., Shigemi A., Takeshita A., Watanabe E., Yokoyama Y., Ikawa K., Morikawa N., Takeda Y. (2014). Analysis of Thrombocytopenic Effects and Population Pharmacokinetics of Linezolid: A Dosage Strategy According to the Trough Concentration Target and Renal Function in Adult Patients. Int. J. Antimicrob. Agents.

[14] Jaspard M., Butel N., El Helali N., Marigot-Outtandy D., Guillot H., Peytavin G., Veziris N., Bodaghi B., Flandre P., Petitjean G. (2020). Linezolid-Associated Neurologic Adverse Events in Patients with Multidrug-Resistant Tuberculosis, France. Emerg. Infect. Dis..

[15] Graciaa D.S., Kipiani M., Magee M.J., Mikiashvili L., Barbakadze K., Bablishvili N., Auld S.C., Alghamdi W.A., Alshaer M.H., Peloquin C.A. (2022). Linezolid Exposure Is Associated with Cytopenias in Patients Treated for Multidrug-Resistant Tuberculosis. Antimicrob. Agents Chemother..

[16] Eimer J., Fréchet-Jachym M., Le Dû D., Caumes E., El-Helali N., Marigot-Outtandy D., Mechai F., Peytavin G., Pourcher V., Rioux C. (2023). Association Between Increased Linezolid Plasma Concentrations and the Development of Severe Toxicity in Multidrug-Resistant Tuberculosis Treatment. Clin. Infect. Dis..

[17] Wasserman S., Brust J.C.M., Abdelwahab M.T., Little F., Denti P., Wiesner L., Gandhi N.R., Meintjes G., Maartens G. (2022). Linezolid Toxicity in Patients with Drug-Resistant Tuberculosis: A Prospective Cohort Study. J. Antimicrob. Chemother..

[18] Deshpande D., Srivastava S., Pasipanodya J.G., Bush S.J., Nuermberger E., Swaminathan S., Gumbo T. (2016). Linezolid for Infants and Toddlers With Disseminated Tuberculosis: First Steps. Clin. Infect. Dis..

[19] Bolhuis M.S., Akkerman O.W., Sturkenboom M.G.G., Ghimire S., Srivastava S., Gumbo T., Alffenaar J.-W.C. (2018). Linezolid-Based Regimens for Multidrug-Resistant Tuberculosis (TB): A Systematic Review to Establish or Revise the Current Recommended Dose for TB Treatment. Clin. Infect. Dis..

[20] Cockcroft D.W., Gault M.H. (1976). Prediction of Creatinine Clearance from Serum Creatinine. Nephron.

[21] Miettinen O.S. (2008). Survival Analysis: Up from Kaplan-Meier-Greenwood. Eur. J. Epidemiol..

[22] Mockeliunas L., Keutzer L., Sturkenboom M.G.G., Bolhuis M.S., Hulskotte L.M.G., Akkerman O.W., Simonsson U.S.H. (2022). Model-Informed Precision Dosing of Linezolid in Patients with Drug-Resistant Tuberculosis. Pharmaceutics.

[23] Keizer R.J., Heine R.t., Frymoyer A., Lesko L.J., Mangat R., Goswami S. (2018). Model-Informed Precision Dosing at the Bedside: Scientific Challenges and Opportunities. CPT Pharmacomet. Syst. Pharmacol..

[24] Cojutti P.G., Merelli M., Bassetti M., Pea F. (2019). Proactive Therapeutic Drug Monitoring (TDM) May Be Helpful in Managing Long-Term Treatment with Linezolid Safely: Findings from a Monocentric, Prospective, Open-Label, Interventional Study. J. Antimicrob. Chemother..

[25] Cattaneo D., Gervasoni C., Cozzi V., Castoldi S., Baldelli S., Clementi E. (2016). Therapeutic Drug Management of Linezolid: A Missed Opportunity for Clinicians?. Int. J. Antimicrob. Agents.

[26] Bolhuis M.S., van der Werf T.S., Kerstjens H.A.M., de Lange W.C.M., Alffenaar J.-W.C., Akkerman O.W. (2019). Treatment of Multidrug-Resistant Tuberculosis Using Therapeutic Drug Monitoring: First Experiences with Sub-300 Mg Linezolid Dosages Using in-House Made Capsules. Eur. Respir. J..

[27] Bolhuis M.S., Akkerman O.W., Sturkenboom M.G.G., de Lange W.C.M., van der Werf T.S., Alffenaar J.-W.C. (2016). Individualized Treatment of Multidrug-Resistant Tuberculosis Using Therapeutic Drug Monitoring. Int. J. Mycobacteriol..

[28] Srivastava S., Magombedze G., Koeuth T., Sherman C., Pasipanodya J.G., Raj P., Wakeland E., Deshpande D., Gumbo T. (2017). Linezolid Dose That Maximizes Sterilizing Effect While Minimizing Toxicity and Resistance Emergence for Tuberculosis. Antimicrob. Agents Chemother..

[29] Cui D., Hu X., Shi L., Wang D., Chen G. (2023). Linezolid-Related Adverse Effects in the Treatment of Rifampicin Resistant Tuberculosis: A Retrospective Study. J. Chemother..

[30] Ouellet D. (2010). Benefit-Risk Assessment: The Use of Clinical Utility Index. Expert. Opin. Drug Saf..

[31] Nyberg J., Karlsson K., Jönsson S., Yin O., Miller R., Karlsson M., Simonsson U. (2016). Edoxaban Exposure-Response Analysis and Clinical Utility Index Assessment in Patients With Symptomatic Deep-Vein Thrombosis or Pulmonary Embolism. CPT Pharmacomet. Syst. Pharmacol..

[32] Alghamdi W.A., Al-Shaer M.H., An G., Alsultan A., Kipiani M., Barbakadze K., Mikiashvili L., Ashkin D., Griffith D.E., Cegielski J.P. (2020). Population Pharmacokinetics of Linezolid in Tuberculosis Patients: Dosing Regimens Simulation and Target Attainment Analysis. Antimicrob. Agents Chemother..

[33] Pea F., Furlanut M., Cojutti P., Cristini F., Zamparini E., Franceschi L., Viale P. (2010). Therapeutic Drug Monitoring of Linezolid: A Retrospective Monocentric Analysis. Antimicrob. Agents Chemother..

[34] Björnsson M.A., Friberg L.E., Simonsson U.S.H. (2014). Performance of Nonlinear Mixed Effects Models in the Presence of Informative Dropout. AAPS J..

[35] Siddiqui O., Hung H.M.J., O’Neill R. (2009). MMRM vs. LOCF: A Comprehensive Comparison Based on Simulation Study and 25 NDA Datasets. J. Biopharm. Stat..

[36] Rubin D.B. (1976). Inference and Missing Data. Biometrika.

[37] Laird N.M. (1988). Missing Data in Longitudinal Studies. Stat. Med..

[38] Pharmacia & Upjohn Company Clinical Pharmacology and Biopharmaceutics Review(s). Zyvox Tablets, I.V. & Oral Suspensions (Linezolid) 1999. https://www.accessdata.fda.gov/drugsatfda_docs/nda/2002/21-130S003_Zyvox_biopharmr.PDF.

[39] Harmelink I.M., Alffenaar J.-W., Wessels A.M.A. (2008). A Rapid and Simple Liquid Chromatography-Tandem Mass Spectrometry Method for the Determination of Linezolid in Human Serum. Eur. J. Hosp. Pharm..

[40] Trotti A., Colevas A.D., Setser A., Rusch V., Jaques D., Budach V., Langer C., Murphy B., Cumberlin R., Coleman C.N. (2003). CTCAE v3.0: Development of a Comprehensive Grading System for the Adverse Effects of Cancer Treatment. Semin. Radiat. Oncol..

[41] Sasaki T., Takane H., Ogawa K., Isagawa S., Hirota T., Higuchi S., Horii T., Otsubo K., Ieiri I. (2011). Population Pharmacokinetic and Pharmacodynamic Analysis of Linezolid and a Hematologic Side Effect, Thrombocytopenia, in Japanese Patients. Antimicrob. Agents Chemother..

[42] Boak L.M., Rayner C.R., Grayson M.L., Paterson D.L., Spelman D., Khumra S., Capitano B., Forrest A., Li J., Nation R.L. (2014). Clinical Population Pharmacokinetics and Toxicodynamics of Linezolid. Antimicrob. Agents Chemother..

[43] Tsuji Y., Holford N.H.G., Kasai H., Ogami C., Heo Y., Higashi Y., Mizoguchi A., To H., Yamamoto Y. (2017). Population Pharmacokinetics and Pharmacodynamics of Linezolid-induced Thrombocytopenia in Hospitalized Patients. Br. J. Clin. Pharmacol..

[44] Pascoalinho D., Vilas M.J., Coelho L., Moreira P. (2011). Linezolid-Related Immune-Mediated Severe Thrombocytopenia. Int. J. Antimicrob. Agents.

[45] Bernstein W.B., Trotta R.F., Rector J.T., Tjaden J.A., Barile A.J. (2003). Mechanisms for Linezolid-Induced Anemia and Thrombocytopenia. Ann. Pharmacother..

[46] Beal S., Sheiner L., Boeckmann A., Bauer R. (1989). NONMEM 7.4 Users Guides.

[47] Carlsson K.C., Savic R.M., Hooker A.C., Karlsson M.O. (2009). Modeling Subpopulations with the $MIXTURE Subroutine in NONMEM: Finding the Individual Probability of Belonging to a Subpopulation for the Use in Model Analysis and Improved Decision Making. AAPS J..

[48] Keizer R.J., Karlsson M.O., Hooker A. (2013). Modeling and Simulation Workbench for NONMEM: Tutorial on Pirana, PsN, and Xpose. CPT Pharmacomet. Syst. Pharmacol..

[49] R Core Team (2015). R: A Language and Environment for Statistical Computing.

