# Supplementary material for: Derivation and Clinical Utility of Safety Targets for Linezolid-Related Adverse Events in Drug-Resistant Tuberculosis Treatment
Source: Pharmaceuticals (Basel). 2023 Nov 8;16(11):1575. doi: 10.3390/ph16111575 (PMC10674798; doi:10.3390/ph16111575)
Supplement: Supplementary file 1 [file pharmaceuticals-16-01575-s001.zip › pharmaceuticals-2687013-supplementary.pdf]

Supplementary Material

Derivation and Clinical Utility of Safety Targets for Linezolid-Related Adverse Events in Drug-Resistant Tuberculosis Treatment

Lina Keutzer [1], Laurynas Mockeliunas [1], Marieke G. G. Sturkenboom [2], Mathieu S. Bolhuis [2], Onno W. Akkerman [3,4] and Ulrika S. H. Simonsson [1,*]

[1] Department of Pharmaceutical Biosciences, Uppsala University, 751 24 Uppsala, Sweden
[2] Department of Clinical Pharmacy and Pharmacology, University Medical Center Groningen, University of Groningen, 9713 GZ Groningen, The Netherlands
[3] Department of Pulmonary Diseases and Tuberculosis, University Medical Center Groningen, University of Groningen, 9713 GZ Groningen, The Netherlands
[4] Tuberculosis Center Beatrixoord, University Medical Center Groningen, University of Groningen, 9751 ND Groningen, The Netherlands
* Correspondence: ulrika.simonsson@uu.se

Supplementary Figures

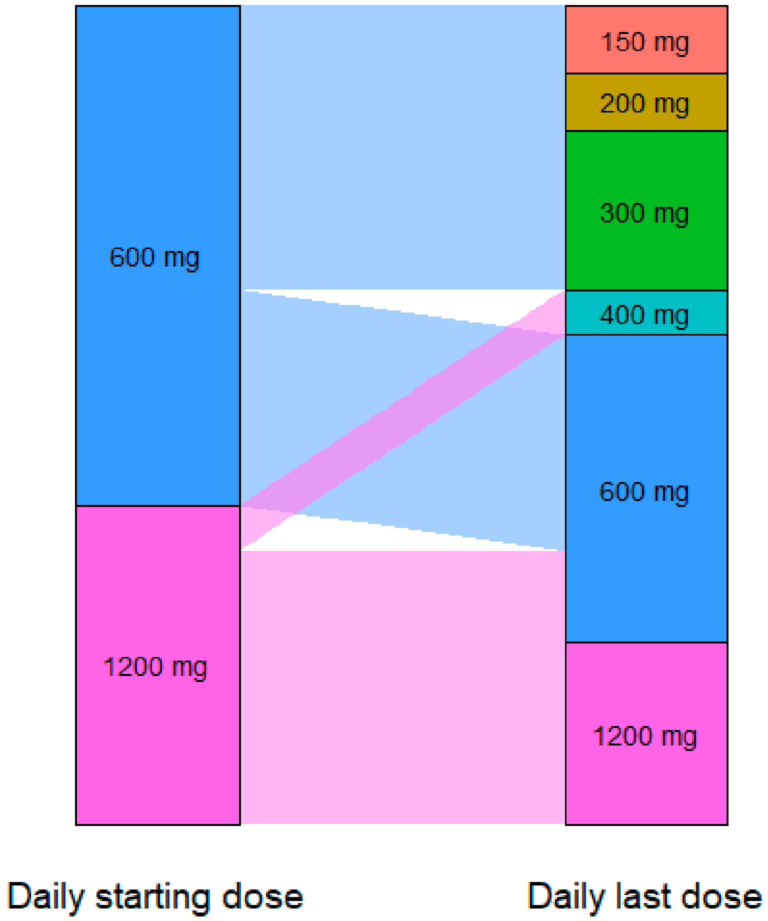

Figure S1. Sankey plot showing individual dose adjustments between start and end of treatment. The size of the boxes represents the proportion of patients falling within the respective category. The lines show the proportion of patients transitioning to another category, here daily dose.

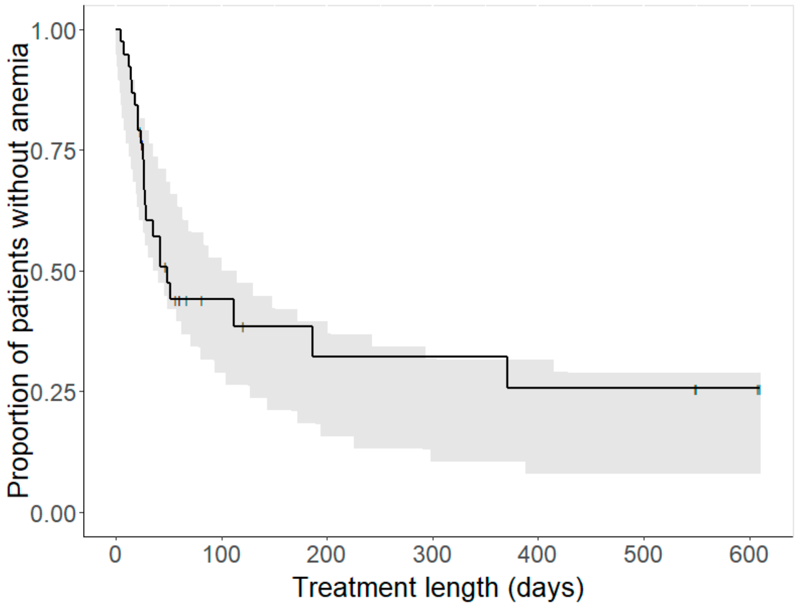

(a)

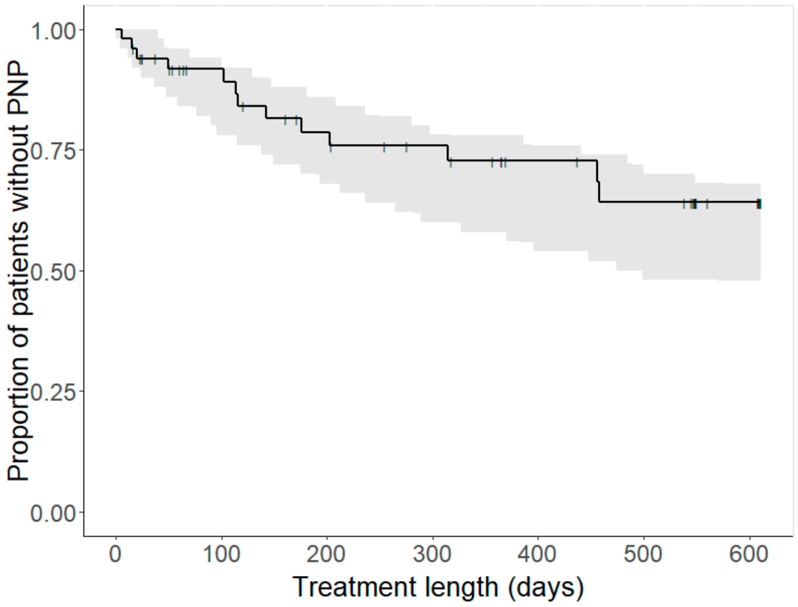

(b)

Figure S2. Kaplan-Meier Visual Predictive Check (1000 samples) of the final linezolid exposure-response model for (**a**) anemia and (**b**) peripheral neuropathy. The solid line represents the Kaplan-Meier estimate of the observed data and the grey shaded area the 95% prediction intervals using the final exposure-response model for each endpoint.

PNP, peripheral neuropathy.

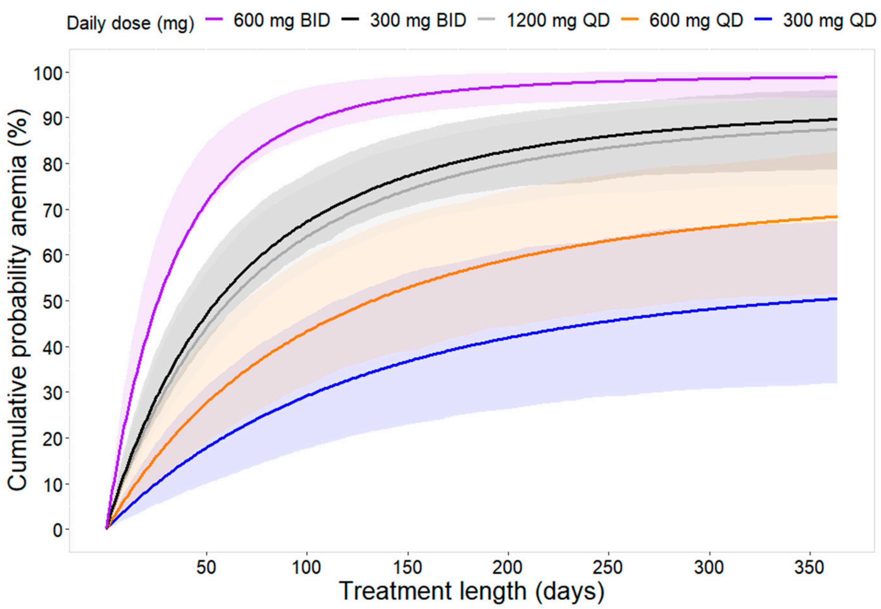

(a)

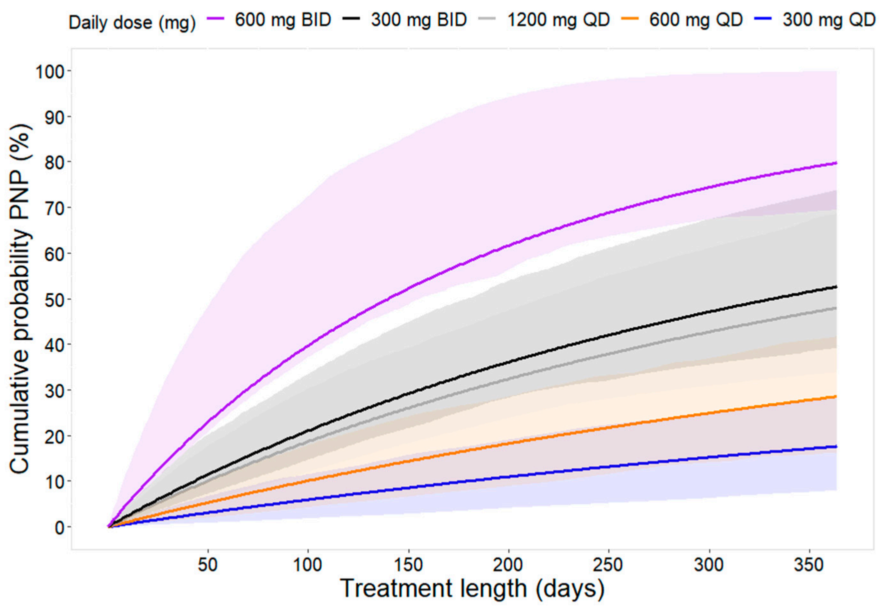

(b)

Figure S3. Cumulative probability of an event at different linezolid exposures versus treatment length for (**a**) anemia and (**b**) peripheral neuropathy. The solid lines represent the median model predictions for each linezolid trough concentration. The different colors indicate the different daily linezolid doses. The shaded areas are the 95% confidence interval given the uncertainty in the model parameters (1000 samples).

PNP, peripheral neuropathy.

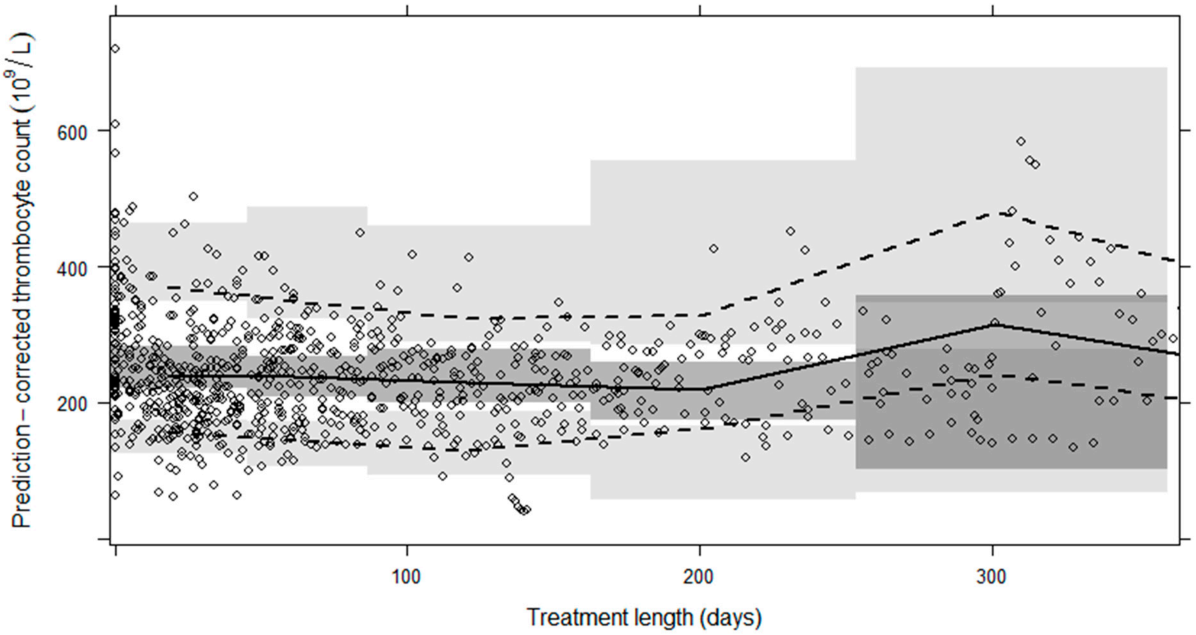

Figure S4. Prediction-corrected visual predictive check for the final thrombocyte indirect response model showing data up to 1 year. Open circles are the prediction-corrected observations. The upper and lower dashed lines are the 90th and 10th percentiles of the observed data, respectively. The solid line is the median of the observed data. The shaded areas (top to bottom) are the 95% confidence intervals of the 95th (light grey), median (dark grey), and 5th (light grey) percentiles of the simulated data based on 1000 simulations.

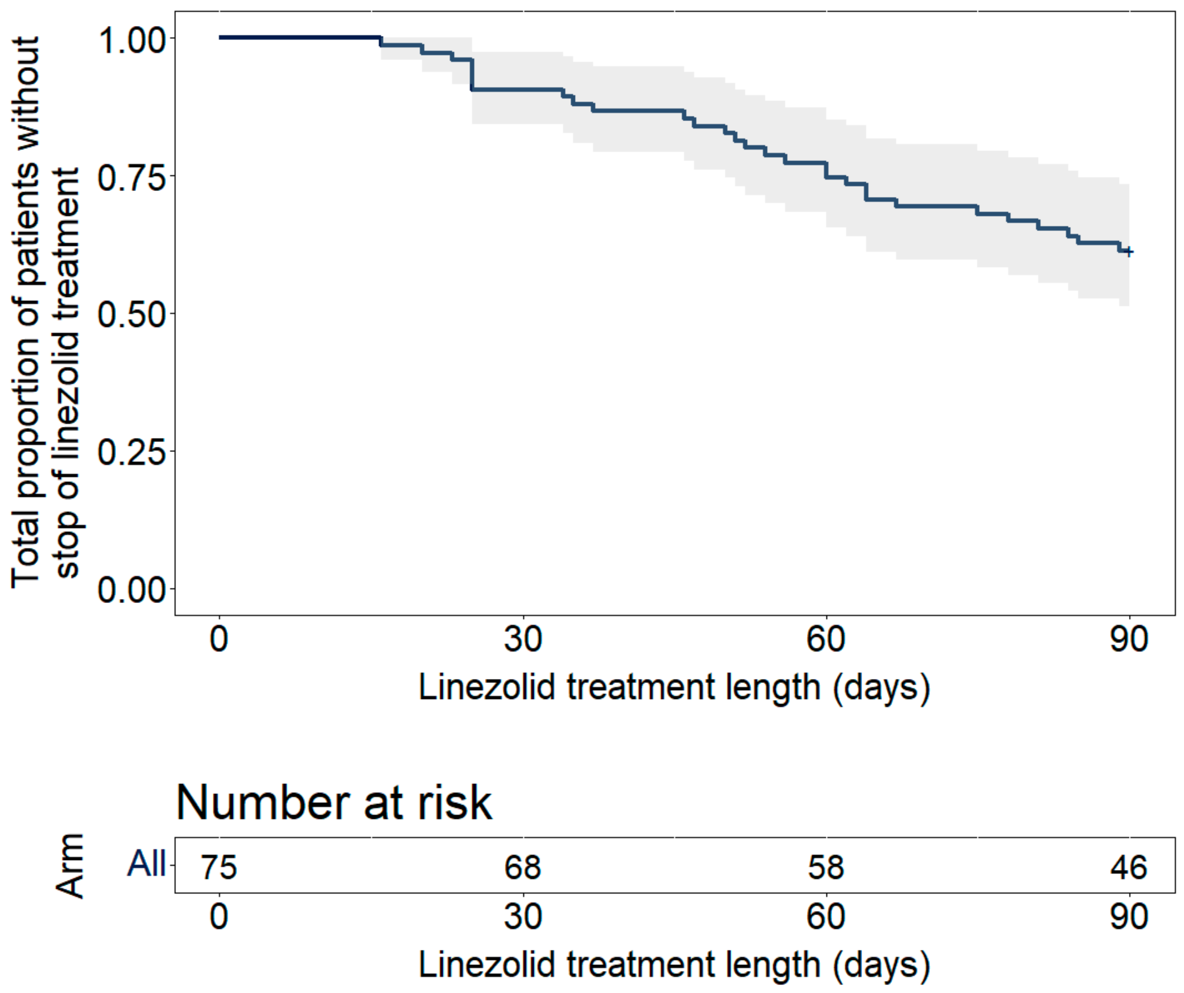

Figure S5. Kaplan-Meier plot for the time to dropout event. The shaded area represents the standard error, computed using the Greenwood method [21]. Vertical dashes represent censoring, while downward steps represent events. The number at risk table represents the total number of participants without the event or censoring.

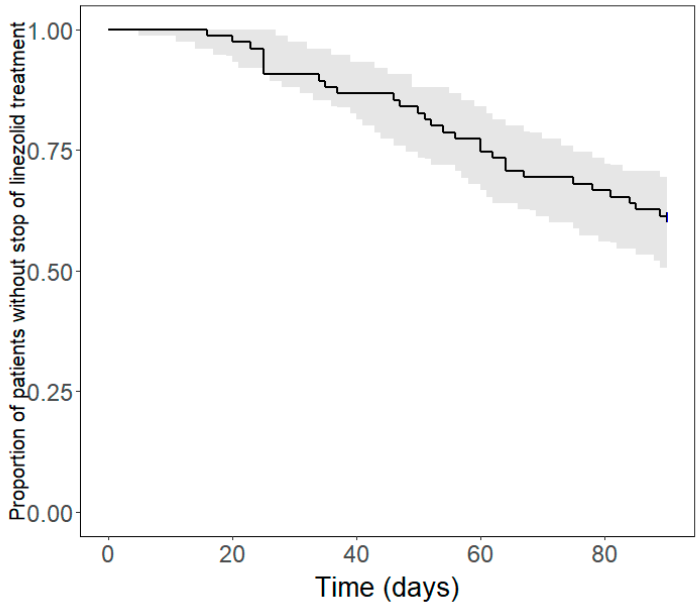

(a)

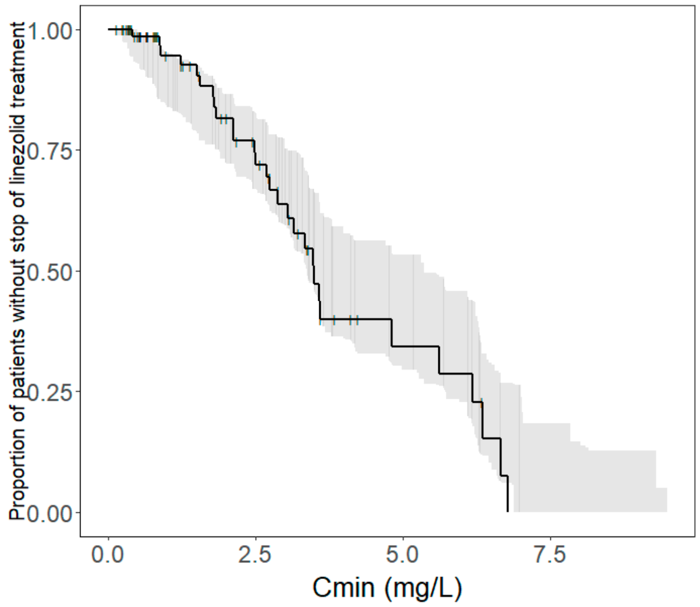

(b)

Figure S6. Kaplan Meier visual predictive check (1000 samples) of the final dropout model showing (**a**) dropout versus time and (**b**) dropout versus linezolid C_{min}. The solid line represents the Kaplan-Meier estimate of the observed data and the grey shaded area the 95% prediction interval using the final exposure-response model.

C_{min}, linezolid trough concentration.

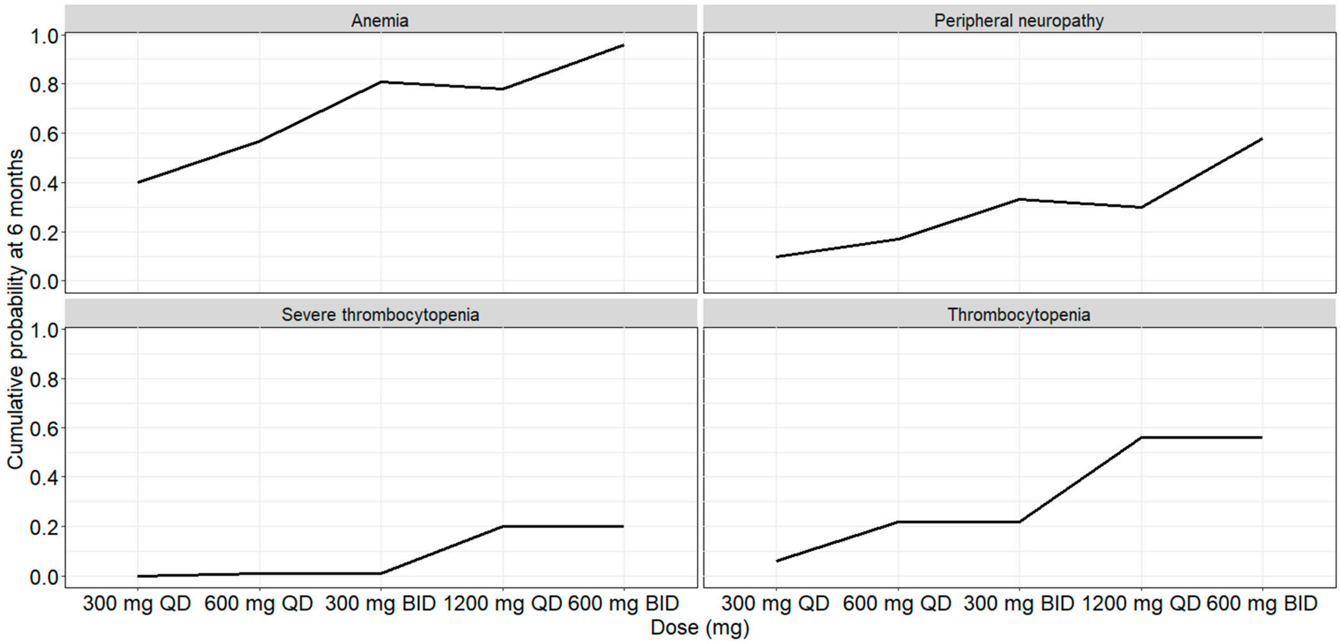

Figure S7. Model-predicted typical cumulative probabilities at 6 months for the four safety endpoints.

PNP, peripheral neuropathy, QD, once daily; BID, twice daily.

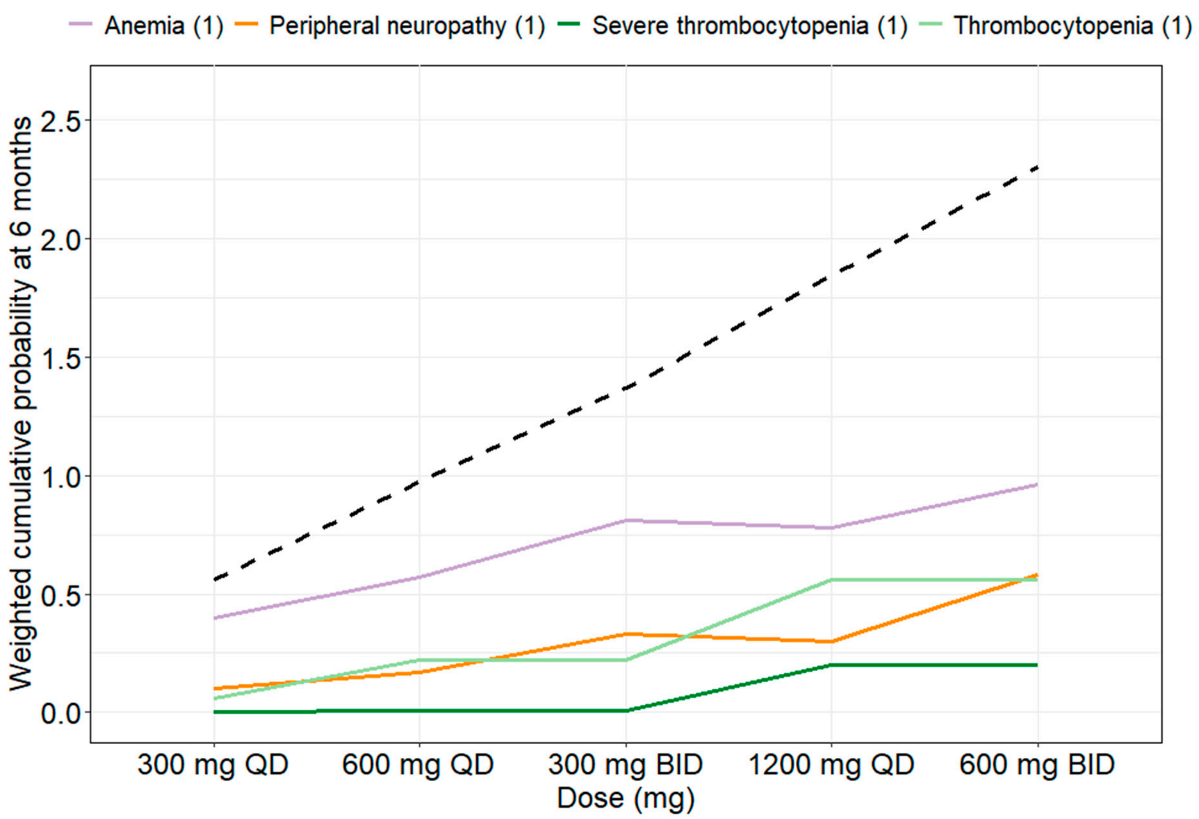

Anemia (1)
Peripheral neuropathy (1)
Severe thrombocytopenia (1)
Thrombocytopenia (1)
Weighted cumulative probability at 6 months
0.0
0.5
1.0
1.5
2.0
2.5
300 mg QD
600 mg QD
300 mg BID
1200 mg QD
600 mg BID
Dose (mg)

(**a**)

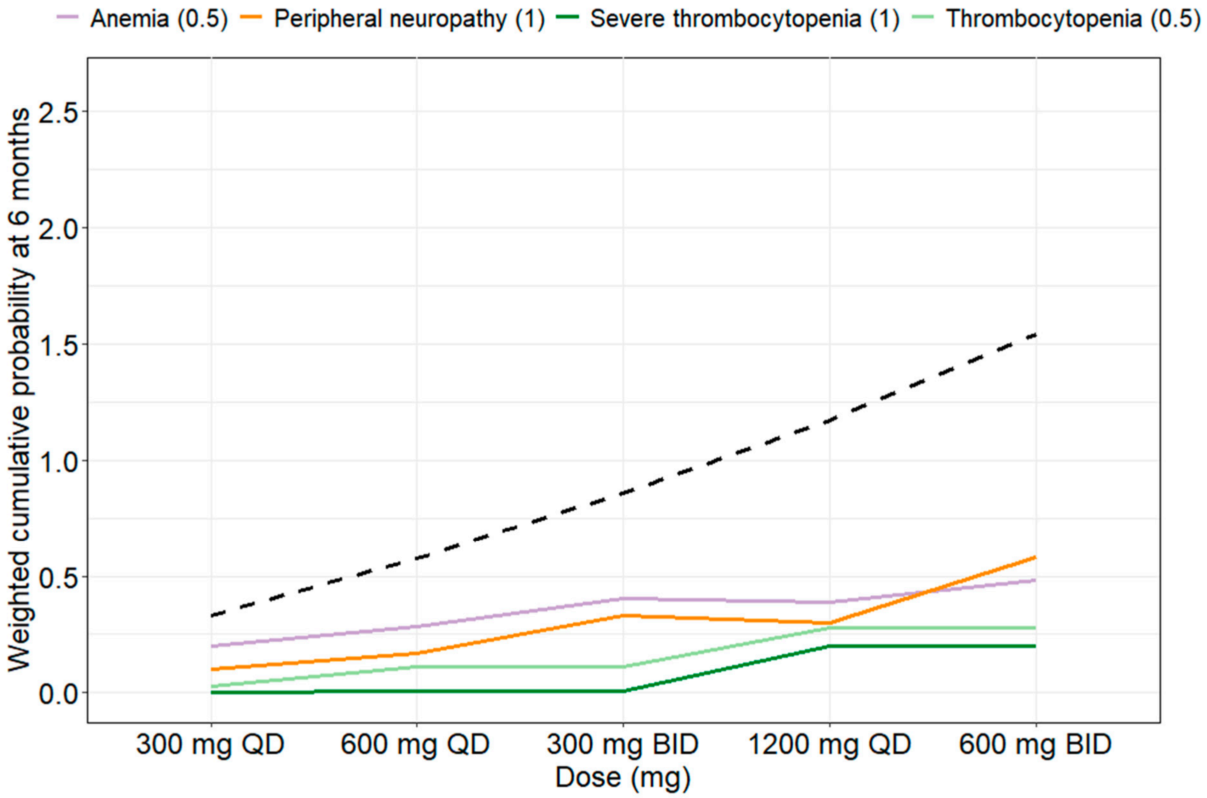

Anemia (0.5)
Peripheral neuropathy (1)
Severe thrombocytopenia (1)
Thrombocytopenia (0.5)
Weighted cumulative probability at 6 months
0.0
0.5
1.0
1.5
2.0
2.5
300 mg QD
600 mg QD
300 mg BID
1200 mg QD
600 mg BID
Dose (mg)

(**b**)

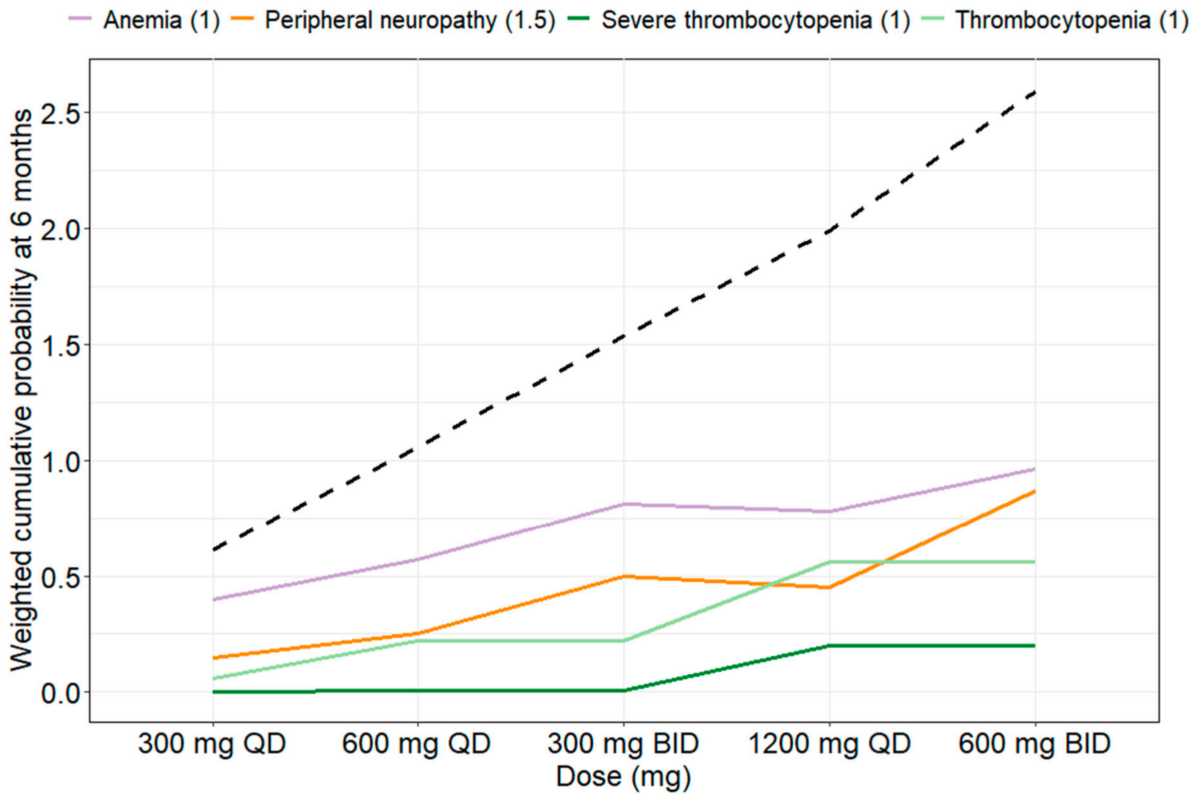

(c)

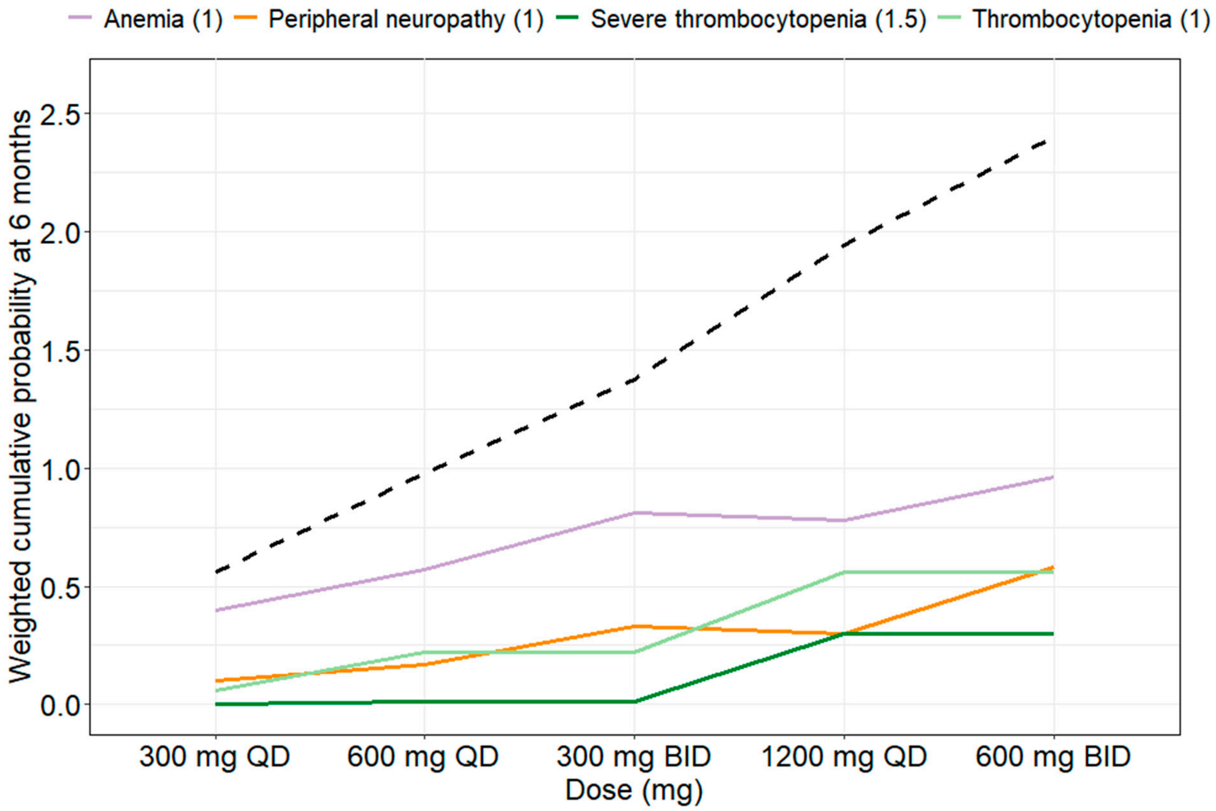

(d)

Figure S8. Sensitivity analysis for the weighting applied to the clinical utility assessment. The different weights for peripheral neuropathy (PNP), anemia, thrombocytopenia and severe

thrombocytopenia are (**a**) 1:1:1:1, (**b**) 1:0.5:0.5:1, (**c**) 1.5:1:1:1 and (**d**) 1:1:1:1.5. The black dashed line indicates the clinical utility index (CUI) derived as the sum of the weighted cumulative probabilities for all four endpoints.

PNP, peripheral neuropathy, QD, once daily; BID, twice daily.

Supplementary Tables

Table S1. Summary of co-medications.

**Co-Medication**	**No. of Patients (%)**
Amikacin	41 (55)
Bedaquiline	6 (8)
Capreomycin	1 (1)
Clofazimine	45 (60)
Clarithromycin	3 (4)
Cycloserine	4 (5)
Co-Trimoxazole	9 (12)
Delamanid	1 (1)
Ertapenem	7 (9)
Ethambutol	39 (52)
Isoniazid	5 (7)
Kanamycin	7 (9)
Moxifloxacin	62 (83)
Protionamide	10 (13)
Pyrazinamide	28 (37)
Rifabutin	2 (3)
Rifampicin	1 (1)
Tigecycline	1 (1)

Table S2. Targets for peripheral neuropathy and severe thrombocytopenia for different target cumulative probabilities.

**Cumulative Probability (%)**	**Treatment Length**	**Target C_{min} Peripheral Neuropathy**	**Target AUC_{0-24h} Severe Thrombocytopenia**
5	6 months	0.15	<172
5	12 months	0.05	<160
10	6 months	0.5	<206
10	12 months	0.2	<195

AUC_{0-24h}, area under the linezolid plasma concentration-time curve up to 24 hours; C_{min}, linezolid trough concentration.

References

21. Miettinen, O.S. Survival Analysis: Up from Kaplan-Meier-Greenwood. *Eur. J. Epidemiol.* **2008**, *23*, 585–592, doi:10.1007/s10654-008-9278-7.
